# Multi-omics revealed the formation mechanism of characteristic volatiles in Tibetan yak cheese induced by different altitudes

**DOI:** 10.1016/j.fochx.2024.101120

**Published:** 2024-01-03

**Authors:** Bei Xue, Guo Li, Xujia Xun, Qun Huang, Shaokang Wang

**Affiliations:** a School of Medicine, Xizang Minzu University, Xianyang, Shanxi 712082, China; b School of Public Health, Guizhou Province Engineering Research Center of Health Food Innovative Manufacturing, the Key Laboratory of Environmental Pollution Monitoring and Disease Control of Ministry of Education, Guizhou Medical University, Guiyang 550025, China; c School of Public Health, Southeast University, Nanjing Jiangsu 210009, China

**Keywords:** Tibetan yak cheese, Altitudes, Volatiles, Microorganism, Metabolomics

## Abstract

The variation in volatiles, bacteria and metabolites of Tibetan yak cheese (TYC) from different altitudes were characterized with multi-omics to reveal the formation mechanism of characteristic volatile compounds (C-VOCs) in TYC induced by altitudes. 22C-VOCs (odor activity value, OAV > 1) were identified in TYCs, and hexanal, dodecanol, 2,3-butanediol, butyl isobutyate, etc., C-VOCs were confirmed induced by altitude. *Lactobacillus*, *Kocuria*, etc., bacteria and benzyl thiocyanate, trehalose, sarcosine, etc., metabolites were screened as the variable bacteria and metabolites for TYCs regulated by altitude, respectively. *Pediococcus* and carbonhydrates maybe the main contributors for the formation of C-VOCs in TYCs induced by altitudes. The formation of dodecanol, 2,3-butanediol and hexanal maybe derived from sarcosine and EPA, and the generation of butyl isobutyrates maybe originated from 1,6-DP-fructose and threonic acid facilitating by *Pediococcus*. This research will help us gain insight into the contribution of altitude to the formation of volatiles in TYCs.

## Highlights

• 22 characteristic volatile compounds (C-VOCs, OAV > 1) were screened in TYCs.
• Hexanal, nonanal, 2-nonanone, etc., C-VOCs in TYCs could be affected by altitude.
• *Lactobacillus, Kocuria*, etc., bacteria in TYCs maybe regulated by altitude.
• Benzyl thiocyanate, trehalose, etc., metabolites in TYCs maybe regulated by altitude.
• Variable metabolites and bacteria correlated to C-VOCs in TYCs induced by altitudes.

## 1 Introduction

Yak cheese, an acid curd hard cheese also called Qula, was one of the most important staple foods for local resident in Tibetan and has been eaten in this area for thousands of years (Pei et al., 2017). Tibetan yak cheese (TYC) was naturally fermented and air-dried from boiled and skimmed yak milk without going through maturity and adding rennet (Zhang et al., 2008). It has long been believed that dietary TYC with higher protein and conjugated linoleic acid could cure gastritis diseases, prevent heart disease and metabolic disorders (Wang et al., 2015).

What's more, TYC was famous for its unique mouth-feel and characteristic flavor due to the balance among volatile and non-volatile compounds (Wang et al., 2015, Song et al., 2015). The volatile compounds of TYC are important part for cheese’s flavor, since some of them are responsible for its characteristic aroma (Song et al., 2015, Wang et al., 2015). It was well known that the formation of volatiles in TYC was influenced by many factors such as milk origin, process technology, starters and others (Song et al., 2015, Wang et al., 2015).

It was well acknowledged that microorganisms play a crucial role in the formation of volatile compounds in fermented foods and diversified microorganisms also maybe increase desirable texture, aroma, and flavor for fermented foods (Wang et al., 2022, Wang et al., 2022, Aziz et al., 2021). Therefore, the diversity of bacterial community in yak milk dreg (Chi et al., 2021) and the effect of starter cultures on the volatiles in yak milk cheese have been reported in recent years (Wang et al., 2015, Song et al., 2015).

To be specific, previous researches have shown that *Lactic acid bacteria* equipped with variable enzyme could improve the volatiles for cheese by regulating the metabolism of amino acids (Teter et al., 2020). The bacterial community of TYC significantly differed among samples from different origins and affected by geography factors, including seasonal variation and other environmental conditions (Chi et al., 2021).

It was common that TYCs obtained at different altitudes with different volatile profiles and contain variable microorganisms. In other words, the composition, abundance, and activity of microorganisms in Alpine forest soils and the volatile profile of endemic diplotaenia bingolensis were reported both regulated or affected by altitude (Halil et al., 2018, Siles et al., 2006). It could be inferred that the difference in volatile profile emitted from TYCs should be realized by adjusting the metabolic activity of microorganisms through altitude. However, to our knowledge, no literature has focused on the variation in volatiles emitted from TYCs caused by altitude.

It was well known that small molecular metabolites were mainly responsible for the formation of volatiles and regulated by microorganism in foods. For example, the generation of isoamyl alcohol could be attributed to cysteine (Hazelwood et al., 2008), and the formation of decanal may depend on the Maillard reaction of proline with glucosamine (Kwak et al., 2004). Moreover, the degradation of keto acids could be regulated by Mortierella alpine (Li et al., 2022). Thereby, it was necessary to explore the metabolic activity of bacteria and the contribution of metabolites on the formation of volatiles in TYCs.

Therefore, the objectives of this manuscripts were (i) to identify the characteristic volatiles in TYCs induced by altitudes; (ii) to filter for differences in bacteria and metabolites induced by altitude; (iii) to investigate the potential relationship between bacteria with characteristic volatiles and metabolites both induced by altitude. At last, (iv) to clarify the formation pathway of characteristic volatiles emitted from TYC induced by altitudes. In a word, the purpose of this study was to investigate the effect of metabolism (pathway) induced by different altitudes on the formation of characteristic volatiles in TYCs.

## 2 Materials and methods

### 2.1 Collection or preparation of Tibetan yak cheeses

Tibetan yak cheeses were collected from the local herdsmen in Tibetan at different altitudes, Nyingchi (LZ), Rikaze (RKZ), Naqu (NQ), using sterile sampling bags and then transported to the laboratory using a foam box filled with dry-ice as soon as possible. The collected samples were stored at 4 °C until further analysis no more than three days. The milk used to prepare cheese came from the mountain yaks aged 5 to 6 years fed with same proportion of grass and grain under similar conditions and then fermented at −4 ∼ 12 °C for two days and ripened for a week. Meanwhile, the other processing techniques and conditions for cheeses were controlled as consistently as possible. The other detailed parameters for sampling were presented in Table S1.

### 2.2 Identification of volatiles in Tibetan yak cheeses

#### 2.2.1 SPME

Briefly, 3.0 g of TYCs from each area was mixed and added into a 20 mL headspace vial (CNW Technologies, Duesseldorf, Germany) and then transferred to a heating furnace pre-balanced 70 °C for 30 min. The volatile compounds emitted form each group of TYC were extracted or collected by 50/30 μm DVB/CAR/PDMS SPME fiber in headspace mode for 45 min and then desorbed in the injection port of GC at 230 °C for 5 min.

#### 2.2.2 GC–MS conditions

The separation and identification of volatiles in TYCs were performed by Clarus 680–600 T GC–MS (Perkin Elmer, USA) equipped with E-WAX ETR (30 m × 0.25 mm, 0.50 μm) in spiltless mode. Helium with high purity (99.99 %) was used as a carrier gas with a constant flow of 1.0 mL/min and the inlet was held at 250 °C. The following temperature program was used: initial temperature 35 °C held for 2 min, and then increasing at a rate of 4 °C/min to 120 °C, increasing to 180 °C at a rate of 6 °C/min, ultimately reached 230 °C at a rate of 10 °C/min and held 10 min.

The temperature of electron impact ionization (EI) source was set at 230 °C and electron impact mode was operated in 70 eV. The mass quad was set at 150 °C and the masses were scanned over a range of 30–500 *m*/*z*.

#### 2.2.3 Qualitative and quantitative analysis of volatile compounds

The volatiles emitted from TYC from each area were tentative identified by matching spectral components to reference standards in NIST 20 MS database and further confirmed by standards. The quantification of these volatiles emitted from TYC were further quantified based upon the standard curves obtained via adding a series levels of representative standards for each class of volatile compounds. Such as hexanal (≥99.0 %), heptanal (≥98.0 %), nonanal (≥96.0 %), 2-octenal (≥98.0 %), 1-pentanol (≥99.5 %), octanoic acid (>95.0 %), 1-octen-3-ol (≥98.0 %), and 3-octen-2-one (≥97.0 %), butyl Isobutyrate (>95.0 %), and so on.

### 2.3 Calculation of OAV

The odor activity value (OAV) were calculated by the concentration of each volatile compounds emitted from TYC dividing by their odor threshold value. Which could reflected the contribution of each component to the overall aroma. It is commonly believed that the compounds with OAV ≥ 1 were considered to be responsible for the overall aroma of foods. The calculation of OAVs were calculated according to the following formula:

OAV = C_i_/OT_i_

where C_i_ is the concentration of each volatile, OT_i_ is the value of odor threshold for each compound (Zhu et al., 2016).

### 2.4 Identification of metabolites in Tibetan yak cheeses

#### 2.4.1 Derivatization of metabolites in Tibetan yak cheeses

Briefly, 50 mg of TYC was mixed with 40 μL internal standard (IS, 0.3 mg/mL, L-2-chloro-phenylalanine in methanol) in 1.5 mL EP tube and then added and mixed thoroughly with 360 μL cold methanol. The mixtures were extracted in a ultrasonic with ice bath for 30 min and then 200 µL of chloroform and 400 µL of pure water were added, respectively. The extracted mixtures were centrifuged at 10,000 r/min for 10 min (4 °C) and 400 µL of dried supernatant were derived with 15 mg methoxyamine, respectively.

#### 2.4.2 Metabolites in Tibetan yak cheeses analyzed by GC–MS

The derivatization of metabolites in TYC from each area (1 µL) were analyzed using 8890B-5977B GC–MS system (Agilent, USA) equipped with HP-5MS column (30 m × 0.25 mm × 0.25 μm) in splitless mode. The inlet was set at 260 °C and helium with high purity (99.99 %) was used as a carrier gas with a constant flow of 1.0 mL/min. The thermal gradient started at 60 °C (4 min for solvent delay) to 310 °C at a rate of 8 °C/min and then held for 6 min. Electron impact at 70 eV was used to ionize the samples, and the MS source and quadrupole were set at 230 °C and 150 °C, respectively. Full mass scan took place from 50 to 600 *m*/*z* and the data were acquired with MSD Chem Station software, USA. The identification of metabolites were performed by comparing its mass spectral feature (matching factor > 800) in the database of NIST 14.0 (National Institute of Standards and Technology, USA) and relative quantification through their peak area (Liu et al., 2023).

### 2.5 Determination of microbiome in Tibetan yak cheeses

The total DNA of microbiome in TYC (0.5 g) were extracted using TPDQeX prep *GEM* universal DNA kit (Kurabo, Japan). The concentration, purity, and integrity of extracted DNA were determined by Nanodrop (Thermo Scientific, Illkirch, France) and checked by 1.5 % agarose gel electrophoresis. The 16S rDNA of bacterial from TYC were amplified using primers 338F (5′-ACTCCTACGGGAGGCAGCAG-3′) and 806R (5′-GGACTACHVGGGTWTCTAAT-3′) purchased from Sangon Biotech Co., Ltd. (Shanghai, China). The PCR amplification products were sequenced on an Illumina Miseq PE300 platform (Biomarker Bioinformatics Technology Co., Ltd, Beijing, China) and sequencing data were performed using quantitative insights into microbial ecology software. Operational taxonomic units (OTUs) with 97 % similarity cutoff were clustered using Mothur v1.30.2 UPARSE (v7.0.1090) and the taxonomy of each OTU sequence was analyzed by RDP Classifier (v2.11).

### 2.6 Statistical analysis

The volatile compounds identified in TYC from three herdsmen for each altitudes were analyzed, quantified and presented as: mean ± SE. The visualization and/or variation presentation for data obtained from TYC were performed principal component analysis (PCA), partial least squares discriminant analysis (OPLS-DA), cluster analysis using Metaboanalyst 5.0. The relationship among volatile compounds, metabolites and microbiome were performed and visualized by Metaboanalyst 5.0 and Cytoscape 3.9.0, respectively.

## 3 Results and discussion

### 3.1 Comparison of characteristic volatiles in TYC from different altitudes

The composition and content of volatiles in TYC obtained with SPME were detected and quantified by GC–MS combined with standard compounds. The characteristic volatiles in TYC from different altitudes were screened and identified by odor activity values (OAVs) and odor description (Table 1). A total of 43 volatile compounds, including 15 alcohols, 9 aldehydes, 8 esters, 7 ketones, etc., were identified in TYC. Most of them, including hexanal, heptanal, octanal, benzaldehyde, 1-pentanol, 2-heptanol, 1-hexanol, 2-nonanol, phenylethyl alcohol, 2-heptanone, 2-pentanone, 2-nonanone, 2-octen-one, 2-undecanone, hexanoic acid, etc., compounds, have been reported in yak milk powder and cheese products (Bertuzzi et al., 2018).

**Table 1:** Comparison of volatiles in Tibetan yak cheese from different altitudes.

Compounds	Odor description	LZ (μg/kg)	RKZ (μg/kg)	NQ (μg/kg)	Odor threshold (μg/kg)	OAV		
						LZ	RKZ	NQ
**Hexanal**	**grass, tallow, fat**	**30 ± 2.45**	**81.67 ± 3.4**	**116 ± 3.27**	**5**	**6 ± 0.49**	**16.33 ± 0.68**	**23.2 ± 0.65**
Heptanal	fat, citrus, rancid	578.33 ± 6.24	–	478 ± 8.16	3	192.78 ± 2.08	–	159.33 ± 2.72
Octanal	fat, soap, lemon, green	168.67 ± 2.05	331.33 ± 2.87	270 ± 2.45	0.7	240.95 ± 2.94	473.33 ± 4.1	385.71 ± 3.5
trans-2-Heptenal	green	40.33 ± 2.49	20 ± 2.16	57 ± 4.55	13	3.1 ± 0.19	1.54 ± 0.17	4.38 ± 0.35
Nonanal	fat, citrus, green	1373.33 ± 3.86	282.33 ± 2.49	518.67 ± 3.68	1	1373.33 ± 3.86	282.33 ± 2.49	518.67 ± 3.68
Decanal	soap,orange,peel,tallow	210.33 ± 2.49	133.33 ± 2.49	159.67 ± 2.05	2	105.17 ± 1.25	66.67 ± 1.25	79.83 ± 1.03
Benzaldehyde	almond, burnt sugar	147 ± 1.63	120.67 ± 3.3	228.33 ± 4.11	350	<1	<1	<1
trans-2-Octenal	fat,	95.33 ± 2.05	65.67 ± 0.94	132.67 ± 3.09	0.08	1191.67 ± 25.69	820.83 ± 11.79	1658.33 ± 38.64
**trans-2-Decenal**	**green,fat,**	**132.67 ± 1.7**	**190.67 ± 2.62**	**67.33 ± 2.05**	**0.3**	**442.22 ± 5.67**	**635.56 ± 8.75**	**224.44 ± 6.85**
**Aldehydes (9)**		**9**	**8**	**9**				
2-Heptanone		–	400 ± 3.27	–	140	–	2.86 ± 0.02	–
**2-Nonanone**	**green,fat,**	**905.33 ± 6.13**	**609.67 ± 4.5**	**300.67 ± 9.84**	**5**	**181.07 ± 1.23**	**121.93 ± 0.9**	**60.13 ± 1.97**
3-Octen-2-one		–	–	46 ± 0.82	–			
8-Octen-2-one		91.33 ± 2.87	30 ± 2.45	56.33 ± 3.68	–			
3,5-Octadien-2-one		45 ± 5.72	20 ± 0.82	169.33 ± 3.3	–			
3,5-Octadien-2-one		80.33 ± 2.87	28 ± 0.82	198 ± 2.45	–			
**2-Undecanone**		**150.33 ± 2.87**	**139.33 ± 4.92**	**–**	**7**	**21.48 ± 0.41**	**19.9 ± 0.7**	**–**
**Ketones (7)**		**5**	**6**	**5**				
Hexanoic acid		191 ± 2.45	240.33 ± 2.36	54.33 ± 3.3	420	<1	<1	<1
**Octanoic acid**		**440.67 ± 3.68**	**410.33 ± 2.36**	**38. ± 3.27**	500	<1	<1	<1
**Decanoic acid**		**402.67 ± 6.24**	**257 ± 1.63**	–	130	3.1 ± 0.05	1.98 ± 0.01	–
**Acids (3)**		**3**	**3**	**2**				
Isoamyl alcohol		115.67 ± 4.5	365.33 ± 4.19	–	250	0.46 ± 0.02	1.46 ± 0.02	–
1-Pentanol		110.33 ± 3.68	81 ± 2.94	333 ± 3.27	4000	<1	<1	<1
2-Heptanol		60 ± 2.16	41.33 ± 1.7	59.33 ± 1.25	100	<1	<1	<1
1-Hexanol		102.67 ± 4.99	191.67 ± 3.3	151 ± 2.94	250	<1	<1	<1
1-Octen-3-ol		–	–	32 ± 0.82	1	–	–	32 ± 0.82
1-Heptanol		66.33 ± 5.31	150.67 ± 3.3	92.33 ± 2.05	3	22.11 ± 1.77	50.22 ± 1.1	30.78 ± 0.68
2-butyl-1-octanol		284 ± 3.74	94 ± 4.55	130.67 ± 2.49	–			
isooctanol		325.67 ± 5.44	–	–	250	1.3 ± 0.02	–	–
2-Nonanol		–	93.67 ± 2.87	83 ± 2.45	–			
2,3-Butanediol		–	255 ± 6.16	177.67 ± 2.87	1000	–	<1	<1
1-Octanol		232.67 ± 2.87	112.33 ± 3.4	236.33 ± 5.31	110	2.12 ± 0.03	1.02 ± 0.03	2.15 ± 0.05
**(R,R)-2,3-Butanediol**		–	**682 ± 4.55**	**1204.3 ± 5.44**	**1000**	–	**0.68 ± 0**	**1.2 ± 0.01**
1-Nonanol		24.67 ± 3.86	27 ± 3.27	66.67 ± 5.31	50	0.49 ± 0.08	0.54 ± 0.07	1.33 ± 0.11
Phenylethyl alcohol		181.33 ± 5.73	371 ± 4.55	254.33 ± 3.68	1000	<1	<1	<1
Dodecanol		–	22.67 ± 1.25	945.33 ± 7.13	–			
**Alcohols (15)**		**10**	**13**	**13**				
Hexanoic acid ethyl ester		50.33 ± 2.49	81 ± 3.27	30.67 ± 2.87	1	50.33 ± 2.49	81 ± 3.27	30.67 ± 2.87
Octanoic acid ethyl ester		68.33 ± 2.05	117.33 ± 3.3	39 ± 2.94	15	4.56 ± 0.14	7.82 ± 0.22	2.6 ± 0.2
Decanoic acid ethyl ester		34.67 ± 1.7	192 ± 2.94	65.33 ± 4.5	500	<1	<1	<1
Phenylethyl acetate		39.67 ± 1.25	143.33 ± 3.3	95.33 ± 3.3	–			
Undecanoicacid ethyl ester		–	110 ± 3.74	–	–			
**Butyl Isobutyrate**		**197 ± 2.45**	**79.67 ± 2.05**	**43 ± 6.98**	**80**	**2.46 ± 0.03**	**1 ± 0.03**	**0.54 ± 0.09**
**Heptyl isobutyrate**		**119.33 ± 3.3**	**74 ± 2.94**	**49.67 ± 2.87**	**13**	**9.18 ± 0.25**	**5.69 ± 0.23**	**3.82 ± 0.22**
Ethyl stearate		–	–	112.67 ± 2.49	–			
**Esters (8)**		**6**	**7**	**7**				
γ-Nonalactone		–	–	23.33 ± 2.05	65	–	–	<1

**Altitudes** LZ:2900 m; RKZ:3800 m; NQ:4500 m; Bold word: indicates the potential compounds related with altitude.

Among them, 22 potential aroma-active compounds (OAV > 1) were screened in TYCs, such as 17, 17 and 16 compounds in LZ, RKZ and NQ, respectively. More importantly, nonanal, octanal, decanal, *trans*-2-octenal, *trans*-2-decenal, 2-nonanone (OAV > 100) with lower threshold or higher concentration may play an important role for the overall flavor of TYCs (Table 1). Therefore, it can be concluded that aliphatic aldehydes may be the characteristic volatiles of TYC from three different altitudes and inferred that the difference in overall flavor should be attributed to the abundance and proportion in them.

### 3.2 Characteristic volatiles in Tibetan yak cheese induced by different altitudes

**Fig. 1:**
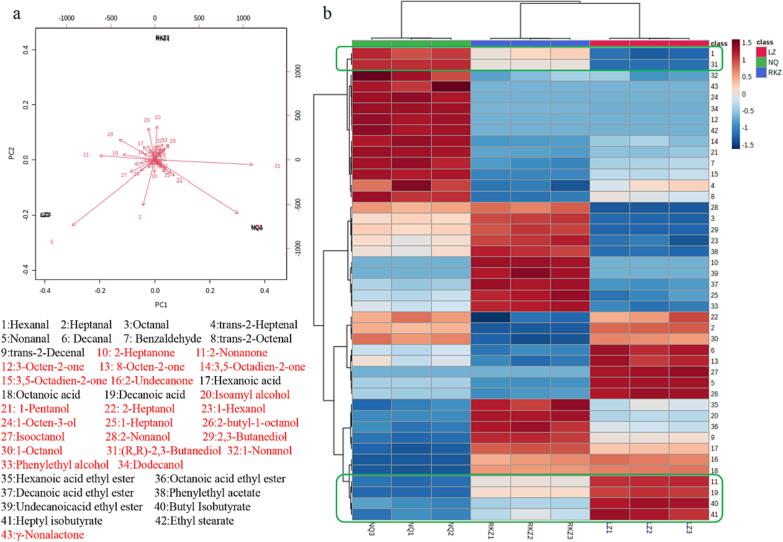
Comparison of volatiles in Tibetan yak cheese from different altitudes (a:bio-polt for PCA & b:heatmap for clustering).

Furthermore, PCA and cluster analysis were used to visualize the difference in volatiles of TYC from different altitudes (Fig. 1). It is obvious that there is a significant difference among the profile of volatiles in TYC from low (LZ), middle (RKZ), and high (NQ) altitude areas (Fig. 1a). The volatiles of TYC from LZ, NQ and RKZ were distributed in the negative and positive direction of PC_2_, respectively. Which can be interpreted as the content of nonanal, heptanal, dodecanol, etc., compounds and 2-heptanone, isoamyl alcohol, octanoic acid, etc., compounds emitted from RKZ were lower and higher than those emitted from LZ and NQ, respectively.

It was well known that nonanal and heptanal, etc., straight-chain aldehydes were derived from the catabolism of fatty acids and which maybe affected by light (Kim et al., 2003) and the homogeneity of milk (Velez et al., 2017). Dodecanol was tentatively identified in may bryndza cheese in Slovakia and affected by environmental conditions, processing methods and so on (Sakova et al., 2015). In addition, it was also reported that 2-heptanone contribute slightly sweet, ester, waxy and fuller aroma to the cheese maybe derived from the catabolism of free fatty acids and affected by commercial starter and yeasts (Velez et al., 2017, Li et al., 2022). The formation of isoamyl alcohol in cheese (products) could been stimulated or promoted by yeast (Li et al., 2022, Li et al., 2022) and octanoic acid was observed to form more easily in homogeneous cheese at the end of ripening (Velez et al., 2017). It can be concluded that the formation of characteristic volatiles in cheeses were regulated and affected by variable factors, such as culture time, temperature, microorganism, and so on.

More importantly, it was also noticed that the volatiles of TYC from LZ, RKZ and NQ were distributed from negative to positive in the direction of PC_1_ (Fig. 1a) and distributed from right to left in the heatmap for clustering (Fig. 1b). This could be attributed to the intensity of (R,R)-2,3-butanediol, hexanal, dodecanol, nonanal, 1-pentanol, etc., compounds and 2-nonanone, octanoic acid, butyl isobutyrate; heptyl isobutyrate, etc., compounds emitted form TYCs obtained from high altitudes (Tibet region: 2900–4500 m) were gradually increased and decreased, respectively (Fig. 1). Therefore, it could be inferred that the variation in these volatiles in TYCs may be mainly regulated or controlled by altitude rather than any other factors.

Fortunately, most of these differential volatiles, including 2,3-butanediol, hexanal, 1-pentanol, and 2-nonanone, isobutyrates (homologous series), etc., compounds, affected by altitudes were identified or reported in cheese products (Cardinali et al., 2021). Meanwhile, 1-pentanol and 2-nonanone were usually considered as the secondary products of lipid oxidation (Andersen et al., 2008) and significantly affected by light-exposure (Mortensen et al., 2002). The generation of 2,3-butanediol from saccharides has been reported to be associated with variable microorganisms (Fernández-Gutiérrez et al., 2017).

What’s more, the contents of oxidation or degradation products from lipids, such as hexanal, nonanal, and 1-pentanol, etc., compounds were observed significantly higher in high altitude jerky (HAJ) than those in low altitude jerky (LAJ) during natural drying. Similarly, 2,3-butanediol from amino acid catabolism and most esters were also found higher in HAJ than those in LAJ during natural drying, especially in the early days. While, the abundance of 2-nonanone was reported to be more likely to form in the earlier period of natural drying rather than later period (Han et al., 2020). So, it can be speculated that the formation of characteristic volatiles in TYCs may be affected by microorganisms (and their metabolites) regulated by altitude.

### 3.3 Variable microorganism in Tibetan yak cheese induced by different altitudes

**Fig. 2:**
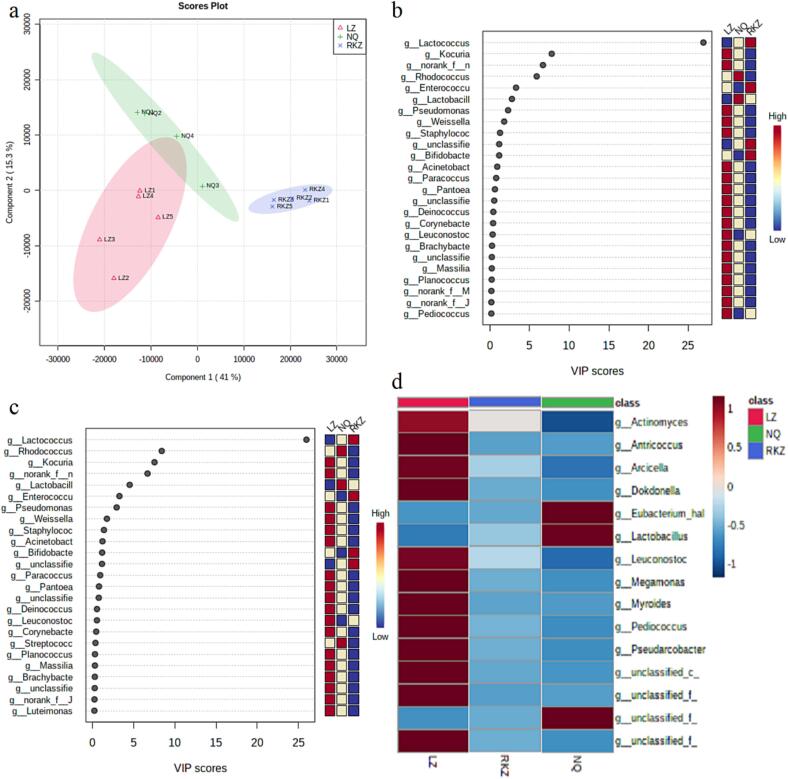
Comparison of bacterium in Tibetan yak cheese from different altitudes (a:score plot for PLS-DA; b and c:VIP for PC_1_ and PC_2_, respectively; d:clustering heatmap for bacteria in TYC induced by altitudes).

Furthermore, PCA (Fig. 2a) and cluster analysis (Fig. 2d) were used to visualize and screen for differential microbes in TYCs. Similarly, the abundance of *Lactobacillus*, *Kocuria*, *Rhodococcus*, etc., bacteria were found to be higher in TYC from middle altitudes (RKZ) than those from high (NQ) or low (LZ) altitude area (Fig. 2a,b). More importantly, it was worth noting that the intensity of *Lactobacillus*, *Eubacterium*, etc., bacteria and *Leuconostoc*, *Actinomyces*, *Arcicella*, etc., bacteria were observed to be positively and negatively correlated with altitude, respectively (Fig. 2c,d).

Fortunately, most of the above differential bacterial communities, including *Lactobacillus*, *Leuconostoc*, *Actinomyces*, etc., have been detected and reported in cheeses or fermented dairy products (Liang et al., 2021). The aroma or taste properties of fermented dairy products were regulated by *Lactobacillus* due to its effect on the degradation of disaccharide lactose, protein, and lipids (Holzapfel & Wood, 2014). The formation of ethyl-esters, and ethanol have been observed correlated with *Leuconostoc* (Dan et al., 2019), and the levels of total terpene was found increased in cheeses at higher altitudes (Turri et al., 2021).

### 3.4 Variable metabolites in Tibetan yak cheese induced by different altitudes

**Fig. 3:**
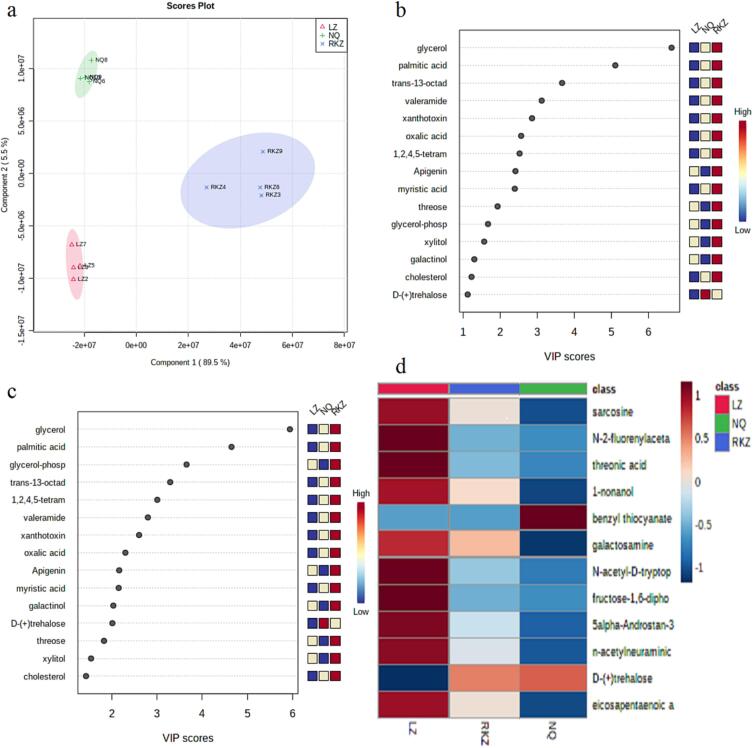
Comparison of metabolites in Tibetan yak cheese from different altitudes. (a:score plot for PLS-DA; b and c:VIP for PC1 and PC2,respectively; d:clustering heatmap for metabolites in TYC induced by altitudes).

It was easy to understand that metabolites were the precursor for volatiles and regulated by microorganism in foods. Thus, the variation of metabolites in TYCs from different altitudes were detected and visualized to filter the (differential) metabolites in TYC induced by different altitudes (Fig. 3). It was obvious that the intensity of glycerol, palmitic acid, *trans*-13-octadecenoic acid, valeramide, xanthotoxin, oxalic acid, apigenin cholesterol, myristic acid, threose, etc., metabolites in TYC from middle altitude (RKZ) was higher than that in TYC from high (NQ) or low (LZ) altitude area (Fig. 3b,c).

Similarly, benzyl thiocyanate, trehalose and sarcosine, N-2-fluorenylacetamide, threonic acid, 1-nonanol, galactosamine, *N*-acetyl-d-tryptophan, fructose-1,6-diphosphate, 5α-androstan-3,7-dione, *n*-acetylneuraminic acid, eicosapentaenoic acid, etc., metabolites were found to be positively and negatively correlated with altitude, respectively (Fig. 3d). Most species of Selaginella survived in high altitude zones maybe due to the presence of trehalose (Chandran & Muralidhara, 2014). Galactosamine-induced hepatitis on energy metabolism could be explained by the shift of aerobic metabolism to anaerobic glycolysis at high altitude (Yamamoto et al., 1995). The levels of sarcosine and eicosapentaenoic acid were found increased and decreased in high-altitude pikas (Cao et al., 2017) and fish liver(Xu et al., 2023). respectively.

### 3.5 Formation mechanism of characteristic volatiles in Tibetan yak cheese induced by different altitudes

#### 3.5.1 Correlation between characteristic volatiles with metabolites and microorganism induced by different altitudes

**Fig. 4:**
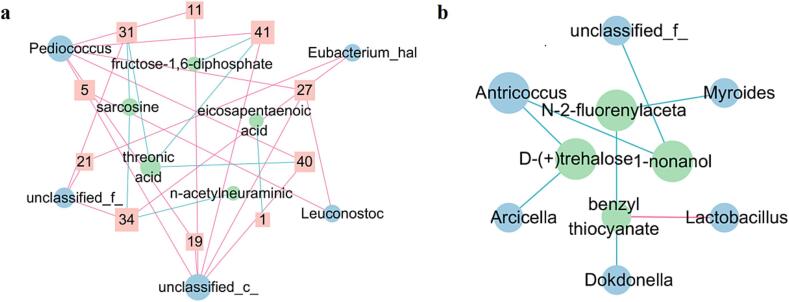
Correlation network among characteristic volatiles, metabolites and microorganism in TYC induced by different altitudes (a:pearson correlation between characteristic volatiles with metabolites and microorganism; b:pearson correlation between metabolites and microorganism; p < 0.05; each node (roundness) represents a microorganism (blue) or a metabolite (green); each brick-red box represents a volatiles; brick-red and blue line: positive and negative correlation) 1:hexanal; 5:nonanal; 11:2-nonanone; 19:decanoic acid; 21:1-pentanol; 27:isooctanol; 31:(R,R)-2,3-butanediol; 34:dodecanol; 40:butyl isobutyrate; 41:heptyl isobutyrate.

Furthermore, correlation (analysis) network were performed and visualized to clarify the relationship between characteristic volatiles with metabolites and microorganism in TYC induced by altitude (Fig. 4). Obviously, 19 positive and 8 negative correlation linkages were observed between 10 characteristic volatiles with 5 species of bacteria and 5 kinds of metabolites, respectively (Fig. 4a). So it could be inferred that the formation of characteristic volatiles in TYC induced by altitudes could be attributed to the variation in metabolic activity of different microorganisms (Zhao et al., 2023, Zhao et al., 2023).

Surprisingly, *Pediococcus* was found positive correlation with nonanal (5), 2-nonanone (11), decanoic acid (19), isooctanol (27), butyl isobutyrate (40), and heptyl isobutyrate (41). While *Eubacterium* and *Leuconostoc* were observed positive correlation with 1-pentanol (21), dodecanol (34) and nonanal (5), isooctanol (27), respectively (Fig. 4a). Fortunately, the generation of nonanal in meat product has been reported to be regulated by *Pediococcus* due to its anti-oxidation properties (Olaoye & Ayodele, 2011). While as, positive correlations were found between *Pedioccoccus* with acetic acid, and *Weissella* with decanoic acid/isopentanol (Chen et al., 2020). Therefore, it was necessary to further explore the mechanism of variation in characteristic volatiles caused by bacteria mediated by altitude.

The potential associations between these characteristic volatiles in TYC induced by altitudes with their precursors (metabolites) are also presented in Fig. 4a. It was obvious that threonic acid and sarcosine were found negative correlation with (R,R)-2,3-butanediol (31), butyl isobutyrate (40), heptyl isobutyrate (41), and (R,R)-2,3-butanediol (31) and dodecanol (34), respectively. 1,6-Diphosphate fructose, eicosapentaenoic acid and *N*-acetylneuraminic were observed negative correlation with heptyl isobutyrate (41), hexanal (1), and dodecanol (34), respectively.

Fortunately, it was generally accepted that the formation of aldehydes, alcohols and esters maybe derived from α-keto acids or glucoses in ham and the degradation of keto acids (or glucoses) were gradually affected or regulated by *Mortierella alpine*, *Aspergillus ruber* and *Penicillium commune*, respectively (Li et al., 2022, Li et al., 2022). Moreover, there are many negative correlations between the variable bacteria and metabolites in TYC induced by altitudes and only one negative correlation link between Lactobacillus with benzyl-thiocyanate was observed (Fig. 4b). It could be further inferred that the formation of other volatiles in TYC should be facilitated or affected by the metabolic activity of bacteria (Weisskopf et al., 2021).

#### 3.5.2 Potential formation pathways of characteristic volatiles in Tibetan yak cheese induced by different altitudes

**Fig. 5:**
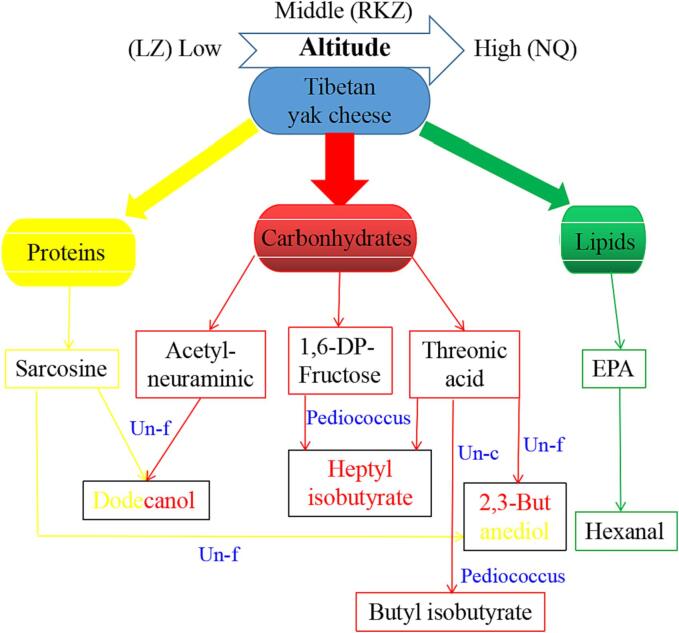
Potential formation pathways of characteristic volatiles in TYC induced by different.

It was suggested that *Pediococcus* and carbonhydrates maybe the main contributors for the formation of characteristic volatiles in TYC induced by altitudes via a variety of biochemical pathways (Fig. 4a & Fig. 5), although the effects of lipids and proteins cannot be ignored. Therefore, the development of characteristic volatiles in TYC induced by altitudes was depicted in the metabolic pathway and visualized in Fig. 5. It was clear that hexanal, one of the characteristic volatiles in TYC induced by altitudes, may be derived from eicosapentaenoic acid (EPA) (Montanari et al., 2018). While, the formation of dodecanol should be attributed to the degradation of sarcosine derived from proteins and regulated by a unclassified bacteria (f) (Fig. 5 & Table S).

More importantly, it was interesting to note that most of these characteristic volatiles induced by altitudes were observed to be derived from carbohydrates with higher content in TYC (Mamet et al., 2022) and mediated primarily by *Pediococcus*. For instance, the formation of butyl isobutyrate was facilitated by unclassified bacteria (c) through threonic acid. While the formation of dodecanol and 2,3-butanediol both derived from the degradation or derivatives of proteins (sarcosine) and could be facilitated by unclassified bacteria (f) (Fig. 5).

What’s more, the generation of dodecanol and 2,3-butanediol could be attributed to acetylneuraminic (Yan et al., 2019) and threonic acid from carbonhydrates in TYC, respectively, and regulated by unclassified bacteria (f). The generation of butyl isobutyrate in TYC maybe promoted by the effect of *Pediococcus* on the degradation of threonic acid from carbonhydrates. Moreover, heptyl isobutyrate maybe generated from threonic acid (Brizzolara et al., 2017) and 1,6-DP-fructose and also been promoted by *Pediococcus* (Yadav and Lathi, 2003)*.*

## 4 Conclusions

A total of 22 characteristic volatiles (OAV > 1), including nonanal, octanal, decanal, 2-octenal, 2-nonanone, etc., compounds were screened in TYCs. Among them, hexanal, nonanal, 2-nonanone, decanoic acid, pentanol, isooctanol, 2,3-butanediol, dodecanol, butyl isobutyate, hepty isobutyrate, etc., compounds could be induced by altitudes. Similarly, the variation in *Lactobacillus*, *Kocuria*, *Rhodococcus*, etc., bacteria and benzyl thiocyanate, trehalose, sarcosine, 2-fluorenylacetamide, threonic acid, nonanol, galactosamine, *N*-acetyl-d-tryptophan, fructose-1,6-diphosphate, eicosapentaenoic acid, etc., metabolites in TYCs were observed to be regulated by altitude.

More importantly, *Pediococcus, Leuconostoc,* etc., bacteria and the degradation or derivatives of carbohydrates (acetylneuraminic, 1,6-DP-fructose and threonic acid), etc., metabolites show significant correlation with these characteristic volatiles in TYC induced by altitudes. More specifically, the formation of dodecanol, 2,3-butanediol and hexanal maybe derived from the degradation or derivatives of proteins (sarcosine) and lipids (EPA), respectively. While, the generation of butyl/ heptyl isobutyrates maybe originated from 1,6-DP-fructose and threonic acid, and facilitated by *Pediococcus*.

In summary, this manuscript focused the effect of altitudes on the formation of characteristic volatiles in TYCs, which would help us understand the contribution of altitudes on the formation of volatiles in fermented products. However, the effects of altitude-induced factors such as humidity, temperature and pressure on volatiles in food could be easily overlooked, and therefore it was necessary to follow up with an in-depth investigation of the effects on altitude-induced oxygen-pressure on the volatiles in fermented products.

## CRediT authorship contribution statement

**Bei Xue:** Writing – review & editing, Writing – original draft, Formal analysis, Data curation. **Guo Li:** Software, Resources, Investigation, Formal analysis, Data curation. **Xujia Xun:** Resources, Investigation, Data curation. **Qun Huang:** Writing – review & editing, Supervision, Project administration. **Shaokang Wang:** Investigation, Data curation, Conceptualization.

## Declaration of competing interest

The authors declare that they have no known competing financial interests or personal relationships that could have appeared to influence the work reported in this paper.

## Data Availability

No data was used for the research described in the article.

## References

[b0005] Andersen C.M., Andersen L.T., Hansen A.M., Skibsted L.H., Petersen M.A. (2008). Wavelength dependence of light-induced lipid oxidation and naturally occurring photosensitizers in cheese. Journal of Agricultural and Food Chemistry.

[b0010] Aziz T., Sarwar A., ud Din J., Al Dalali S., Khan A.A., Din Z.U., Yang Z. (2021). Biotransformation of linoleic acid into different metabolites by food derived Lactobacillus plantarum 12–3 and in silico characterization of relevant reactions. Food Research International.

[b0015] Bertuzzi A.S., McSweeney P.L., Rea M.C., Kilcawley K.N. (2018). Detection of volatile compounds of cheese and their contribution to the flavor profile of surface-ripened cheese. Comprehensive Reviews in Food Science and Food Safety.

[b0020] Brizzolara S., Santucci C., Tenori L., Hertog M., Nicolai B., Stürz S., Zanella A., Tonutti P. (2017). A metabolomics approach to elucidate apple fruit responses to static and dynamic controlled atmosphere storage. Postharvest Biology & Technology.

[b0025] Cao X.F., Bai Z.Z., Ma L., Ma S., Ge R.L. (2017). Metabolic alterations of Qinghai-Tibet plateau pikas in adaptation to high altitude. High Altitude Medicine & Biology.

[b0030] Cardinali F., Ferrocino I., Milanović V., Belleggia L., Corvaglia M.R., Garofalo C., Aquilanti L. (2021). Microbial communities and volatile profile of Queijo de Azeitão PDO cheese, a traditional Mediterranean thistle-curdled cheese from Portugal. Food Research International.

[b0035] Chandran G., Muralidhara (2014). Insights on the neuromodulatory propensity of Selaginella (Sanjeevani) and its potential pharmacological applications. CNS & Neurological Disorders-Drug Targets.

[b0040] Chen C., Liu Y., Tian H., Ai L., Yu H. (2020). Metagenomic analysis reveals the impact of JIUYAO microbial diversity on fermentation and the volatile profile of Shaoxing-jiu. Food Microbiology.

[b0045] Chi F., Tan Z., Gu X., Yang L., Luo Z. (2021). Bacterial community diversity of yak milk dreg collected from Nyingchi region of Tibet, China. LWT-Food Science and Technology.

[b0050] Dan T., Ren W., Liu Y., Tian J., Liu W. (2019). Volatile flavor compounds profile and fermentation characteristics of milk fermented by Lactobacillus delbrueckii subsp. bulgaricus. Frontiers in Microbiology.

[b0055] Fernández-Gutiérrez D.F., Veillette M., Giroir-Fendler A., Ramirez A.A., Faucheux N., Heitz M. (2017). Biovalorization of saccharides derived from industrial wastes such as whey: A review. Reviews in Environmental Science & Bio/Technology.

[b0060] Halil G.I., Ibrahim D., Lutfi B. (2018). Influence of altitude on chemical variability of volatile profile for endemic diplotaenia bingolensis. Chemistry of Natural Compounds.

[b0070] Han G., Zhang L., Li Q., Wang Y., Chen Q., Kong B. (2020). Impacts of different altitudes and natural drying times on lipolysis, lipid oxidation and flavor profile of traditional Tibetan yak jerky. Meat Science.

[bib216] Hazelwood L.A., Daran J.M., Van Maris, Pronk J.T., Dickinson J.R. (2008). The Ehrlich pathway for fusel alcohol production: a century of research on Saccharomyces cerevisiae metabolism. Applied and environmental microbiology.

[b0075] Holzapfel W.H., Wood B.J.B. (2014). Lactic Acid Bacteria.

[b0080] Kim G.Y., Lee J.H., Min D.B. (2003). Study of light-induced volatile compounds in goat's milk cheese. Journal of Agricultural and Food Chemistry.

[bib217] Kwak E.J., Lim S.I. (2004). The effect of sugar, amino acid, metal ion, and NaCl on model Maillard reaction under pH control. Amino acids.

[b0085] Li Y., Wang T., Li S., Yin P., Sheng H., Wang T. (2022). Influence of GABA-producing yeasts on cheese quality, GABA content, and the volatilome. LWT-Food Science & Technology.

[b0090] Li Z., Wang Y., Pan D., Geng F., Zhou C., Cao J. (2022). Insight into the relationship between microorganism communities and flavor quality of Chinese dry-cured boneless ham with different quality grades. Food Bioscience.

[b0095] Liang T., Xie X., Zhang J., Ding Y., Wu Q. (2021). Bacterial community and composition of different traditional fermented dairy products in China, South Africa, and Sri Lanka by high-throughput sequencing of 16S rRNA genes. LWT-Food Science and Technology.

[b0100] Liu Z., Huang Y., Kong S., Miao J., Lai K. (2023). Selection and quantification of volatile indicators for quality deterioration of reheated pork based on simultaneously extracting volatiles and reheating precooked pork. Food Chemistry.

[b0105] Mamet T., Xu B., Li X., Zhang J., Li C., Wang L. (2022). Chemical and nutritional composition of Pamir yak milk from Xinjiang. Journal of Animal Physiology and Animal Nutrition.

[b0110] Montanari C., Gatto V., Torriani S., Barbieri F., Bargossi E., Lanciotti R., Grazia L., Magnani R., Tabanelli G., Gardini F. (2018). Effects of the diameter on physicochemical, microbiological and volatile profile in dry fermented sausages produced with two different starter cultures. Food Bioscience.

[b0115] Mortensen G., Rensen J.S., Stapelfeldt H. (2002). Light-induced oxidation in semihard cheeses. Evaluation of methods used to determine levels of oxidation. Journal of Agricultural & Food Chemistry.

[b0120] Olaoye, Ayodele O. (2011).

[b0125] Pei J., Li X., Han H., Tao Y. (2017). Purification and characterization of plantaricin slg1, a novel bacteriocin produced by Lb. plantarum isolated from yak cheese. Food Control.

[b0130] Sakova N., Sadecka J., Lejkova J., Puskarova A., Korenova J., Kolek E. (2015). Characterization of may bryndza cheese from various regions in Slovakia based on microbiological, molecular and principal volatile odorants examination. Journal of Food & Nutrition Research.

[b0135] Siles J.A., Tomas C., Stefano M., Rosa M. (2006). Effect of altitude and season on microbial activity, abundance and community structure in alpine forest soils. FEMS Microbiology Ecology.

[b0140] Song X., Liang Q., Zhang W., Zhang Y., Ma Y. (2015). Influence of coagulant concentrate on volatile components of hard cheese made from Yak Milk. International Power, Electronics & Materials Engineering Conference..

[b0145] Teter A., Barlowska J., Krol J., Brodziak A., Rutkowska J., Litwinczuk Z. (2020). Volatile compounds and amino acid profile of short-ripened farmhouse cheese manufactured from the milk of the white-backed native cow breed. LWT-Food Science and Technology.

[b0150] Turri F., Cremonesi P., Battelli G., Severgnini M., Pizzi F. (2021). High biodiversity in a limited mountain area revealed in the traditional production of historic rebel cheese by an integrated microbiot-lipidomic approach. Scientific Reports.

[b0155] Velez M.A., Wolf V.I. (2017). Cheese milk low homogenization enhanced early lipolysis and volatiles compounds production in hard cooked cheeses. Food Research International.

[b0160] Wang J., Liu X., Li S., Ye H., Luo W., Huang Q., Geng F. (2022). Ovomucin may be the key protein involved in the early formation of egg-white thermal gel. Food Chemistry.

[b0165] Wang J., Zheng Z., Zhao X., Yang Y., Yang Z. (2015). Effect of starter cultures on the ripening properties of yak milk cheese. Food Science & Technology Research.

[b0170] Wang Q., Li X., Xue B., Wu Y., Song H., Luo Z., Shang P., Liu Z., Huang Q. (2022). Low-salt fermentation improves flavor and quality of sour meat: Microbiology and metabolomics. LWT-Food Science and Technology.

[b0175] Weisskopf L., Schulz S., Garbeva P. (2021). Microbial volatile organic compounds in intra-kingdom and inter-kingdom interactions. Nature Reviews Microbiology.

[b0180] Xu W., Xu W., Zhu F., Wang D., Chen D., Duan X., Liu M., Li D. (2023). Comparative analysis of metabolites between different altitude Schizothorax nukiangensis (Cyprinidae, Schizothoracine) on the Qinghai-Tibet Plateau in Nujiang River. Water.

[b0185] Yadav G.D., Lathi P.S. (2003). Kinetics and mechanism of synthesis of butyl isobutyrate over immobilised lipases. Biochemical Engineering Journal.

[b0190] Yamamoto C., Mori S., Murakami K., Yoshino M. (1995). Effect of galactosamine-induced hepatitis on the aerobic and anaerobic metabolism of the rat exposed to high-altitude hypoxia. Comparative Biochemistry and Physiology Part C: Pharmacology, Toxicology & Endocrinology.

[b0195] Yan Q., Robert S., Brooks J.P., Fong S.S. (2019). Metabolic characterization of the chitinolytic bacterium Serratia marcescens using a genome-scale metabolic model. BMC Bioinformatics.

[b0200] Zhang H., Xu J., Wang J., Menghebilige, Sun T., Li H. (2008). A survey on chemical and microbiological composition of kurut, naturally fermented yak milk from Qinghai in China. Food Control.

[b0205] Zhao H., Ali U., Ren Q., Yao M., Lai T., Naz S., Yang Z. (2023). Integrated metabolomic analysis of Lactiplantibacillus plantarum NMGL2 reveals its survival and response to combinational cold and acidic conditions during storage of fermented milk. Food Bioscience.

[b0210] Zhao M., Wang M., Zhao Y., Hu N., Wang G., Jiang M. (2023). Variations in microbial carbon metabolic activities in sedge peatlands along an altitudinal gradient in the Changbai Mountain, China. Catena.

[b0215] Zhu J., Chen F., Wang L. (2016). Characterization of the key aroma volatile compounds in cranberry (Vaccinium macrocarpon Ait.) using gas chromatography-olfactometry (GC-O) and odor activity value (OAV). Journal of Agricultural & Food Chemistry.

