# Supplementary material for: Multi-omics revealed the formation mechanism of characteristic volatiles in Tibetan yak cheese induced by different altitudes
Source: Food Chem X. 2024 Jan 3;21:101120. doi: 10.1016/j.fochx.2024.101120 (PMC10825365; doi:10.1016/j.fochx.2024.101120)
Supplement: Supplementary data 1 [file mmc1.docx]

**Table S1 Collection information for Tibetan yak cheese (TYC)**

| Tibetan yak  cheese | Herdsmen | Sampling position in Tibet | Coordinates | Temperature | Relative  Humidity | Altitude |
| --- | --- | --- | --- | --- | --- | --- |
| **LZ** | LZ_1_,LZ_2_,LZ_3_ | Milin County, Nyingchi City | E: 94.30° & N: 29.26° | -2-13℃ | 72±5% | **2956 m** |
| **RKZ** | RKZ_1_,RKZ_2_,RKZ_3_ | Bainang County, Rikaze City | E: 89.26° & N: 29.10° | -4-12℃ | 12±10% | **3898 m** |
| **NQ** | NQ_1_,NQ_2_,NQ_3_ | Naqu County, Naqu City | E: 92.00° & N: 31.36° | -3-16℃ | 52±16% | **4516 m** |

Sampling: the TYC obtained from three herdsmen in a (altitude) area were mixed and marked as LZ_1_, LZ_2_, LZ_3_, RKZ_1_,RKZ_2_,RKZ_3_, and NQ_1_, NQ_2_, NQ_3_, respectively. All TYCs from different altitudes were collected in March 2021.

**Table S2 Two important PCs for volatiles in Tibetan yak cheese from different altitudes**

| **Volatiles** | **PC1** | **PC2** | **Volatiles** | **PC1** | **PC2** |
| --- | --- | --- | --- | --- | --- |
| **1** | 0.04 | 0.00 | **23** | 0.03 | 0.05 |
| **2** | -0.07 | **-0.38** | **24** | 0.01 | -0.01 |
| **3** | 0.06 | 0.09 | **25** | 0.02 | 0.06 |
| **4** | 0.01 | -0.03 | **26** | -0.08 | -0.09 |
| **5** | **-0.46** | **-0.54** | **27** | -0.14 | -0.10 |
| **6** | -0.03 | -0.04 | **28** | 0.04 | 0.04 |
| **7** | 0.04 | -0.06 | **29** | 0.08 | 0.12 |
| **8** | 0.02 | -0.04 | **30** | 0.00 | -0.11 |
| **9** | -0.03 | 0.08 | **31** | **0.55** | -0.04 |
| **10** | 0.01 | **0.28** | **32** | 0.02 | -0.02 |
| **11** | **-0.31** | 0.03 | **33** | 0.04 | 0.13 |
| **12** | 0.02 | -0.02 | **34** | **0.47** | **-0.44** |
| **13** | -0.02 | -0.04 | **35** | -0.01 | 0.04 |
| **14** | 0.06 | -0.08 | **36** | -0.01 | 0.06 |
| **15** | 0.06 | -0.10 | **37** | 0.02 | 0.12 |
| **16** | -0.06 | 0.05 | **38** | 0.03 | 0.06 |
| **17** | -0.07 | 0.11 | **39** | 0.00 | 0.08 |
| **18** | **-0.20** | **0.17** | **40** | -0.08 | -0.03 |
| **19** | -0.18 | 0.04 | **41** | -0.04 | -0.01 |
| **20** | -0.04 | **0.27** | **42** | 0.05 | -0.04 |
| **21** | **0.11** | -0.13 | **43** | 0.01 | -0.01 |
| **22** | 0.00 | -0.02 |  |  |  |

1:hexanal; 2:heptanal; 3:octanal; 4:trans-2-heptenal 5:nonanal; 6:decanal; 7:benzaldehyde; 8:trans-2-octenal; 9:trans-2-decenal; 10: 2-heptanone; 11:2-nonanone; 12:3-octen-2-one; 13:8-octen-2-one; 14:3,5-octadien-2-one; 15:3,5-octadien-2-one; 16:2-undecanone;

17:hexanoic acid; 18:octanoic acid; 19:decanoic acid; 20:isoamyl alcohol; 21:1-pentanol; 22:2-heptanol; 23:1-hexanol; 24:1-octen-3-ol; 25:1-heptanol; 26:2-butyl-1-octanol; 27:isooctanol; 28:2-nonanol; 29:2,3-butanediol; 30:1-octanol; 31:(R,R)-2,3-butanediol; 32:1-nonanol; 33:phenylethyl alcohol; 34:dodecanol; 35:hexanoic acid ethyl ester;

36:octanoic acid ethyl ester; 37:decanoic acid ethyl ester; 38:phenylethyl acetate; 39:undecanoicacid ethyl ester; 40:butyl isobutyrate; 41:heptyl isobutyrate; 42:ethyl stearate; 43: γ-Nonalactone
